# Characterization of Leucocin B-KM432Bz from *Leuconostoc pseudomesenteroides* Isolated from Boza, and Comparison of its Efficiency to Pediocin PA-1

**DOI:** 10.1371/journal.pone.0070484

**Published:** 2013-08-01

**Authors:** Kahina Maya Makhloufi, Alyssa Carré-Mlouka, Jean Peduzzi, Carine Lombard, Carol Ann van Reenen, Leon Milner Theodore Dicks, Sylvie Rebuffat

**Affiliations:** 1 Communication Molecules and Adaptation of Microorganisms (MCAM), UMR 7245 CNRS-MNHN, Muséum National d’Histoire Naturelle, Paris, France; 2 Department of Microbiology, University of Stellenbosch, Stellenbosch, South Africa; Teagasc Food Research Centre, Ireland

## Abstract

A bacteriocin-producing bacterium was isolated from boza and identified as *Leuconostoc pseudomesenteroides* KM432Bz. The antimicrobial peptide was purified and shown to be identical to other class IIa bacteriocins: leucocin A from *Leuconostoc gelidum* UAL-187 and *Leuconostoc pseudomesenteroides* QU15 and leucocin B from *Leuconostoc carnosum* Ta11a. The bacteriocin was named leucocin B-KM432Bz. Leucocin B-KM432Bz gene cluster encodes the bacteriocin precursor (*lcnB*), the immunity protein (*lcnI*) and the dedicated export machinery (*lcnD* and *lcnE*). A gene of unknown and non-essential function (*lcnC*), which is interrupted by an insertion sequence of the IS30 family, is localized between *lcnB* and *lcnD*. The activity of leucocin B-KM432Bz requires subunit C of the EII^t^_Man_ mannose permease, which is the receptor for entry into target cells. The determination of the minimum inhibitory concentrations revealed the lowest values for leucocin B-KM432Bz over *Listeria* strains, with 4 to 32 fold better efficiency than pediocin PA-1.

## Introduction

Boza is a traditional popular fermented beverage widely consumed in the Balkan region. It is produced from fermented cereals, such as maize, wheat or millet, mixed with sugar or saccharin [1]. Fermentation is initiated by inoculating homemade natural populations of lactic acid bacteria and yeast originating from boza, or fermented milks, to result in a viscous, low-alcohol, acidic beverage with high nutritional value [2]. Several studies have addressed the microbiology of boza. The main species present belong to the genera *Lactobacillus*, *Lactococcus, Leuconostoc* and *Pediococcus*, many of them being producers of antimicrobial compounds, which are presumed to increase the shelf life of the beverage [3]–[6]. Strains isolated from boza showed mostly antibacterial activity against various Gram-positive bacteria, including *Listeria innocua*, and Gram-negative bacteria such as *Escherichia coli*. Todorov *et al.* [7] reported on the probiotic properties of eight strains isolated from boza, including survival in conditions simulating the gastrointestinal tract, and production of antibacterial and antiviral compounds.

The term antimicrobial compounds refers to a heterogeneous group of chemicals with similar activities, among which bacteriocins are defined as ribosomally synthesized peptides produced by bacteria and exhibiting antagonism against species being phylogenetically related [8], [9]. Bacteriocins may be divided into two main classes based on genetic and biochemical characteristics [10]. Class I consists of the lanthionine-containing post-translationally modified bacteriocins [11]. Class II includes unmodified non-lanthionine-containing bacteriocins, which are subdivided into four subclasses namely, class IIa (pediocin-like bacteriocins), class IIb (two-peptide bacteriocins), class IIc (cyclic bacteriocins) and class IId (non-pediocin single linear bacteriocins) [12]. Bacteriocins have been purified mostly from lactic acid bacteria isolated from different food matrices such as fermented products, vegetables, fruits, meats, and fishes. Potential biotechnological applications of bacteriocins produced by lactic acid bacteria include their use as food preservatives, as they are natural inhabitants of fermented foods and usually inhibit pathogenic bacteria such as *Listeria* or *Enterococcus* [13]–[15]. Nisin, a class I bacteriocin, has been approved for utilization as a preservative in many foods by the European Union in 1983 and the US Food and Drug Agency in 1988 [16] and is commercially available as Nisaplin™ and Chrisin™ [15]. Gene clusters involved in bacteriocin synthesis generally consist of a gene encoding a bacteriocin precursor, which is further cleaved to yield the active peptide, gene(s) involved in affording immunity to the producing strain, gene(s) encoding the export of the active peptide, and gene(s) involved in regulation and production of the peptide. Genes encoding post-translational modification enzymes are often also present [10], [16]. More than twenty bacteriocins have been described for lactic acid bacteria isolated from boza, but only a few have been characterized. Bacteriocin B14, produced by *Lactococcus lactis* subsp. *lactis* 14, was the first to be reported [17]. Mesentericin ST99, produced by *Leuconostoc mesenteroides* subsp. *dextranicum* is heat and acid stable with bacteriostatic activity against *L. innocua* [18]. Other bacteriocins, produced by strains of *Pediococcus pentosaceus*, *Lactobacillus plantarum*, *Lactobacillus paracasei*, *Lactobacillus rhamnosus*, *Lactobacillus pentosus*, *Lactococcus lactis*, *Leuconostoc lactis*, and *Enterococcus faecium*, have been reported from boza [4]–[6], [19], [20]. In most cases, the spectrum of activity, the sensitivity to physico-chemical conditions (temperature, pH) and the production kinetics have been determined only, together with preliminary purification of the bacteriocins. The molecular masses of bacteriocins from lactic acid bacteria found in boza range from 2.8 to 14 kDa, but the amino acid sequences, genetic clusters and modes of action have not been determined for any of these peptides [5].

In this study we describe leucocin B-KM432Bz, a class IIa bacteriocin produced by *Leuconostoc pseudomesenteroides* KM432Bz isolated from boza. The spectrum of antibacterial activity of the purified bacteriocin was determined and the gene cluster involved in production of the active peptide is described. The antibacterial activity of purified leucocin B-KM432Bz is compared to that of pediocin PA-1, a class IIa bacteriocin produced by *Pediococcus acidilactici*, which has been widely studied and has shown good potential as a food preservative [14], [16].

## Materials and Methods

### Bacterial Strains

*Escherichia coli* DH5α was provided by A.-M. Pons (Université de la Rochelle, France). *E. coli* strains were cultivated in LB (Luria-Bertani Broth) at 37°C. Strain KM432Bz was isolated from boza as described previously [5] and registered at the CRBIP (Institut Pasteur, Paris) as CIP110558. *Ent. faecium* HKLHS was obtained from the Department of Microbiology (Stellenbosch, South Africa). *Leuc. mesenteroides* Y105 and *Listeria monocytogenes* EGDe *rpoN ^-^* were provided by Yann Héchard (Université de Poitiers, France). *Listeria monocytogenes* AML73 and the EII^t^_Man_ mannose permease mutants (Δ*manL* and Δ*manM*) were provided by Josef Deutscher and Eliane Milohanic (Institut Micalis, Grignon, France). The other strains were obtained from CRBIP. Strains used in this study are listed in Table 1, together with their culture conditions.

**Table 1:** Inhibitory spectrum of cell-free culture supernatant of *Leuc. pseudomesenteroides* KM432Bz against various target strains.

Target strain	Culture conditions^a^	Inhibition^b^
*Bacillus subtilis* subsp. *subtilis* CIP 52.65	37°C, LB	–
*Enterococcus faecalis* CIP 103015	37°C, BHI	+
*Enterococcus faecium* CIP 103014	37°C, BHI	–
*Enterococcus faecium* HKLHS	37°C, BHI	+
*Klebsiella pneumoniae* subsp. *pneumoniae* CIP 82.91	37°C, LB	–
*Lactobacillus plantarum* CIP 104454	30°C, MRS	–
*Lactobacillus sakei* subsp. *sakei* CIP 103139	30°C, MRS	+
*Leuconostoc carnosum* CIP 103319	30°C, MRS	–
*Leuconostoc citreum* CIP 103315	30°C, MRS	–
*Leuconostoc gelidum* CIP 103318	30°C, MRS	–
*Leuconostoc lactis* CIP 102422	30°C, MRS	+
*Leuconostoc mesenteroides* Y105	30°C, MRS	+
*Leuconostoc mesenteroides* subsp. *dextranicum* CIP 102423	30°C, MRS	+
*Leuconostoc mesenteroides* subsp. *mesenteroides* CIP 102305	30°C, MRS	+
*Leuconostoc pseudomesenteroides* CIP 103316	30°C, MRS	+
*Leuconostoc pseudomesenteroides* KM432Bz	30°C, MRS	–
*Listeria innocua* CIP 80.11	37°C, BHI	+
*Listeria ivanovii* subsp. *ivanovii* CIP 78.42	37°C, BHI	+
*Listeria monocytogenes* CIP 82.110	37°C, BHI	+
*Listeria monocytogenes* EGDe CIP 107776	37°C, BHI	+
*Listeria monocytogenes* EGDe (*rpoN ^-^*)	37°C, BHI	–
*Listeria monocytogenes* AML73	37°C, BHI	+
*Listeria monocytogenes* AML73 Δ*manL*	37°C, BHI	+
*Listeria monocytogenes* AML73 Δ*manM*	37°C, BHI	–
*Staphylococcus aureus* subsp. *aureus* CIP 4.83	37°C, LB	–
*Streptococcus pneumoniae* CIP 102911	37°C, TSYE	+
*Streptococcus thermophilus* CIP 102303	37°C, LB	–
*Weissella paramesenteroides* CIP 102421	30°C, MRS	+

a LB, Luria-Bertani broth; MRS, de Man Rogosa Sharpe; BHI, Brain heart infusion; TSYE, Trypticase soy yeast extract,

b Inhibition halos were measured by radial diffusion assays (+: Inhibition halo; -: No inhibition).

### Identification of Strain KM432Bz

Carbohydrate fermentation reactions of strain KM432Bz were determined using API50CHL strips (Biomérieux, Marcy-l’Etoile, France). Results were compared to the carbohydrate fermentation reactions listed by Farrow *et al.* [21] and those obtained for *Leuc. mesenteroides* subsp. *mesenteroides* CIP102305, *Leuc. mesenteroides* subsp. *dextranicum* CIP102423, *Leuc. mesenteroides* subsp. *cremoris* CIP103009, and *Leuc. pseudomesenteroides* CIP103316. The almost full-length 16S rRNA gene was amplified using eubacterial universal primers 27F and 1385R [22]. Sequencing of the amplified fragments was performed as described below. A phylogenetic tree, based on 16S rRNA gene sequence data, was constructed using http://www.phylogeny.fr/ [23]. Multiple alignments were generated using ClustalW [24].

### Radial Diffusion Assays

After overnight culture in appropriate medium (Table 1), the target strain was inoculated and growth recorded by measuring optical density readings at 620 nm (OD_620nm_). Upon reaching OD_620nm = _0.2, bacterial growth was inhibited by incubation on ice. Melted soft agar (6.5 g/l agar) was inoculated with 10^7^ Colony Forming-Units/ml (CFU/ml) of this culture, and poured into sterile Petri dishes. Five µl of a heated (80°C, 10 min) cell-free culture supernatant of strain KM432Bz or 10 µM purified bacteriocin KM432Bz were spotted onto the surface of the medium and incubated for 24–48 hours at the appropriate temperature for the target strain (Table 1). The diameter of each inhibition zone was recorded. All experiments were repeated in triplicate.

### Determination of Minimal Inhibitory Concentrations (MIC)

To determine the MIC, 10 µM stock solutions of purified bacteriocin KM432Bz or commercial pediocin PA-1 (P0098, Sigma) were prepared in 25 mM ammonium acetate buffer pH 6.5. Serial two-fold dilutions were prepared in the same buffer.

Microtiter plate wells were filled with 90 µl of the appropriate medium and then inoculated with an overnight culture of a sensitive strain (final cell numbers of 10^5^ CFU/ml). In the case of *Listeria* strains final cell numbers of 5.10^4 ^CFU/ml were used. Ten µl of purified bacteriocin KM432Bz or commercial pediocin PA-1 were added at final concentrations ranging from 4 to 1024 nM. After 12–15 hours incubation, the OD_620nm_ was measured using a Multiskan FC microplate photometer (Thermo Scientific, France). The MIC is defined as the lowest bacteriocin concentration for which no growth could be observed. All MIC determinations were performed in triplicate.

### Bacteriocin Production

Production kinetics of bacteriocin KM432Bz was monitored during growth. One hundred ml of MRS broth was inoculated with an overnight culture of strain KM432Bz at a final OD_620nm = _0.1, and incubated at 30°C without agitation. Growth was monitored by following OD_620nm_ for 32 hours. Two ml of culture were collected after 5, 9, 13, 16, 19, 24, 27 and 30 hours of incubation. Following centrifugation at 8,000 g for 10 min at 4°C, the resulting supernatant was heated at 80°C for 10 min. The pH was adjusted to 6.5 using 1 M NaOH. Radial diffusion assays were performed as described previously, using *Lact. sakei* subsp. *sakei* CIP 103139 as target strain. The average diameters of the inhibition zones measured for three independent experiments were determined.

For bacteriocin production, two liters of MRS broth were inoculated with an overnight culture of strain KM432Bz at OD_620nm = _0.1. Following 17–19 hours of incubation at 30°C without agitation, culture supernatants were separated from bacterial cells by centrifugation at 8,000 g for 15 min at 4°C.

### Bacteriocin Purification

Purification was performed from the cell-free supernatant prepared as described above. The supernatant was heated at 80°C for 10 min and the pH adjusted to 6.5 using 1 M NaOH. Ammonium sulphate was gently added to the supernatant to obtain 60% saturation at 20°C. After centrifugation at 25,000 g for 30 min at 4°C, the pellet was solubilized in 200 ml of Milli-Q® water, and dialysed against Milli-Q® water (Spectra-Por®3, MWCO 3.5 kDa) at 4°C for 20 hours with gentle agitation. The resulting solution was freeze-dried and resuspended in 37.5 ml of 25 mM ammonium acetate buffer pH 6.5. The protein solution was then subjected to solid-phase extraction on a SepPak C18 cartridge (10 g, Waters Corp.) previously washed with 200 ml of 80% (v/v) isopropanol in 25 mM ammonium acetate buffer pH 6.5. After a washing step with 200 ml of 25 mM ammonium acetate buffer pH 6.5, successive elution steps were performed with 250 ml of 20, 30, 40, 50 and 60% (v/v) isopropanol in the same buffer. The bacteriocin-containing fraction was identified by radial diffusion assays, as described above, using 5 µl of each fraction and testing against *Lactobacillus sakei* subsp. *sakei* CIP 103139. The active fraction was freeze-dried and resuspended in 250 µl of 10% acetonitrile (ACN) in 25 mM ammonium acetate buffer pH 6.5.

Reversed-phase HPLC (RP-HPLC) was done on an Inertsil ODS2 column (5 µm, 4.6×250 mm/Interchim, France), using a gradient of 0–100% ACN in 0.1% aqueous trifluoroacetic acid (TFA) for 30 min at a flow rate of 1 ml/min. OD_226nm_ was monitored, and fractions were collected manually and tested by radial diffusion assays, as described previously. Fractions containing the purified bacteriocin KM432Bz were concentrated under vacuum in a SpeedVac concentrator (Savant). The bacteriocin KM432Bz concentration was determined using the molar extinction coefficient at 280 nm (14105 M^−1^ cm^−1^) deduced from the amino acid content.

### SDS-PAGE and Gel Overlay

The molecular mass of bacteriocin KM432Bz was estimated by SDS-polyacrylamide gel electrophoresis (SDS-PAGE). Five µl of purified bacteriocin KM432Bz were loaded onto two lanes of a 16.5% SDS-tricine polyacrylamide gel. Following separation, a first part of the gel containing the prestained molecular weight marker (Spectra Multicolor Low Range Protein Ladder, Fermentas) and the purified bacteriocin was fixed in 40% methanol, 13.5% formaldehyde and stained by silver nitrate. The second part of the gel was fixed overnight in 15% isopropanol, 10% acetic acid, and washed with Milli-Q® water for 4 hours. This gel was then deposited on MRS agar, and overlaid by MRS soft agar inoculated with 10^6^ CFU/ml of *Lact. sakei* subsp. *sakei* CIP 103139. Plates were incubated at 30°C until a bacterial lawn was visible and examined for an inhibition halo at the position of the bacteriocin band.

### Mass Spectrometry (MS)

The purity of commercial pediocin PA-1 and the molecular mass of purified bacteriocin KM432Bz were determined by matrix-assisted laser desorption ionization time-of-flight mass spectrometry (MALDI-TOF-MS) on a Voyager-De-Pro MALDI-TOF mass spectrometer (Applied Biosystems) operated in linear positive mode, using α-cyano-4-hydroxycinnamic acid as the matrix. The mass spectrometer was calibrated externally with a peptide mixture (calibration mixture 1, Applied Biosystems). The precise molecular mass of bacteriocin KM432Bz was obtained with electrospray ionization (ESI) source qQ-TOF hybrid mass spectrometer (Q-Star Pulsar, Applied Biosystems) operating in the positive detection mode.

### Edman N-terminal Sequencing

Purified bacteriocin KM432Bz was subjected to reduction and alkylation of the cysteine residues by acrylamide [25]. Dithiotreitol (DTT) was added at a final concentration of 1 M to 180 µl of 20 µM purified bacteriocin KM432Bz. Alkylation was performed in the dark for 1 hour at 37°C in a final volume of 1200 µl, with acrylamide (Sigma) at a final concentration of 2 M. The mixture was loaded on a PVDF membrane. N-terminal sequencing was carried on a Procise 492 automatic protein sequencer (Applied Biosystems) at the microsequencing platform of Institut Pasteur, France.

### LC-MS-MS

Purified bacteriocin KM432Bz was subjected to reduction and alkylation as described by Bulet et al. [26]. Fourty µl of purified bacteriocin KM432Bz at 20 µM were mixed with 40 µl of 2 mM EDTA, 6 M guanidine hydrochloride in 0.5 M Tris-HCl pH 7.5. Two µl of 4 M DTT were added, and the samples were incubated for 1 hour at 45°C under nitrogen. Following addition of 4 µl of 0.5 M 4-vinylpyridine (Sigma), the sample was further incubated in the dark for 10 min at 45°C. The bacteriocin was separated from reagents on Zip-Tip C18 (Omix, Varian). The reduced and alkylated bacteriocin (25 µl) was then subjected to digestion by endoproteinase GluC (Roche) at a final concentration of 20 ng/µl in 100 mM NH_4_HCO_3_ pH 8, and incubated for 16 hours at 25°C. The reaction was stopped by acidification with 0.1% formic acid (FA) and the reaction mixture was desalted on Zip-Tip C18 before MS analysis.

The bacteriocin digest was analyzed by LC-MS/MS on a Dionex U3000 micro-HPLC system connected to the qQ-TOF mass spectrometer (Q-Star Pulsar, Applied Biosystems). The separation was achieved on a Zorbax 300 SB C8 column (3.5 µm, 150×1 mm, Agilent). The elution gradient was 0 to 60% ACN in 0.1% TFA over 20 min at a flow rate of 40 µl/min. Detection wavelength was set at 226 nm and ESI source was operated in positive mode with a spray voltage of 100 eV.

### Determination of the Sequence of the Genes involved in the Production of Bacteriocin KM432Bz

Total DNA was extracted from a 5 ml MRS overnight culture, using UltraClean® microbial DNA isolation kit (MoBio).

PCR reactions were carried out using 1 U Taq DNA polymerase (Promega), 100 nM of each primer (forward and reverse), 10 µM of dNTP *(*Promega), 20 nM MgCl_2_ *(*Promega) in 1X buffer (Promega) and 1 µl of extracted DNA. The primers used in this study are listed in Table 2. The following program was used: 95°C 5 min followed by 35 cycles of 95°C for 30 s, 48–60°C for 30 s and 72°C for 3 min; followed by an extension step of 5 min at 72°C on a Veriti Thermal Cycler (Applied Biosystems). The amplified products were separated on 0.5–1% (w/v) agarose gel in Tris Acetate EDTA buffer containing 1X GelRed® (Biotium, VWR) and visualized under UV illumination. The molecular weight markers were 1 kbp DNA Ladder (0.5 to 10 kbp, NEB) and 100 bp DNA Ladder (0.1 to 1.51 kbp, NEB).

**Table 2:** Primers used for PCR and DNA sequencing.

Primer name	Sequence (5'–3')	Gene/Plasmid
M13F	TTTTCCCAGTCACGACG	pGEM-T Easy Vector
M13R	ACACAGGAAACAGCTATGACC	pGEM-T Easy Vector
lcnI-R	GCCATGTATTGGCCGTTT	*lcnI* (Leucocin B-KM432Bz immunity protein)
lcnB-F	AACATGAAATCTGCGGATAA	*lcnB* (Leucocin B-KM432Bz precursor)
lcnB-R	ACCAGAAACCATTTCCACCA	*lcnB* (Leucocin B-KM432Bz precursor)
lcnBC-F	TTGCATACCCTGTCTCCA	*lcnB-lcnC*
tpase-F2	GGTAAAAGACGTGGTGATGT	*tra8*
tpase-R1	GACCACGTAGATGACGTT	*tra8*
lcnC-F1	CAGGCCGACATGTCCTA	*lcnC*
lcnC-F2	GCGTGTTGCGTACTTC	*lcnC*
lcnC-R	GAAGTACGCAACACGC	*lcnC*
lcnCD-R	CCTTCCATGTCAGTTTTGGC	Intergenic region *lcnC*-*lcnD*
lcnD-F1	TTTACGGACGCTAATGCC	*lcnD* (ABC transporter)
lcnD-F2	TGAATAATGACCAAATGCAA	*lcnD* (ABC transporter)
lcnD-R	CTATTCAGCGCTTTAATCGT	*lcnD* (ABC transporter)
lcnE-R1	AAGGAAAATTGCGGTAACG	*lcnE* (accessory factor)
lcnE-F1	CGTTACCGCAATTTTCCAA	*lcnE* (accessory factor)
lcnE-R2	CATAGCACTTTGGCTGGAA	*lcnE* (accessory factor)
lcnE-R3	TAGCCTGAATCGTCGC	*lcnE* (accessory factor)
27F	CGGTGTGTCAAGGCCC	16S ribosomal RNA
1385R	GAGTTTGATCCTGGCTCAG	16S ribosomal RNA

Amplicons generated by PCR were purified using the GeneClean® Turbo kit (MPBio), and cloned into the pGEM-T Easy vector (Promega) following the manufacturer’s instructions. Plasmid DNA from positive clones was extracted using the QuickLyse® kit (Qiagen) and sequenced by Eurofins MWG Operon (Germany). Sequences were assembled, and analysed using the BLASTN program [27] at the National Center for Biotechnology Information (NCBI, http://blast.ncbi.nlm.nih.gov/).

### Nucleotide Sequence Accession Numbers

The nucleotide sequences were deposited in the Genbank database under accession number JQ904468 (16S rRNA gene) and JQ904469 (leucocin B-KM432Bz gene cluster).

## Results

### Identification of Strain KM432Bz

Strain KM432Bz was isolated from boza and selected as a potential bacteriocin-producing strain for its inhibitory activity over *Lact. sakei* DSM 20017 and LMG 13558 [5]. The almost full-length gene encoding 16S rRNA was amplified using primers 27F and 1385R, and the resulting amplicon (1512 bp) was sequenced on both strands. BLASTN analysis assigned strain KM432Bz to the genus *Leuconostoc*, with the best scores (99.7%) for both *Leuc. pseudomesenteroides* and *Leuc. mesenteroides*. The carbohydrate fermentation profile of strain KM432Bz was compared with those recorded for several closely related *Leuconostoc* strains and was found similar to that of *Leuc. pseudomesent*eroides CIP103316. The ClustalW multiple alignment of 16S rRNA genes of *Leuconostoc* and the resulting phylogenetic tree confirmed that strain KM432Bz belongs to the species *Leuc. pseudomesenteroides* (data not shown).

### Bacteriocin Production Kinetics

**Figure 1:**
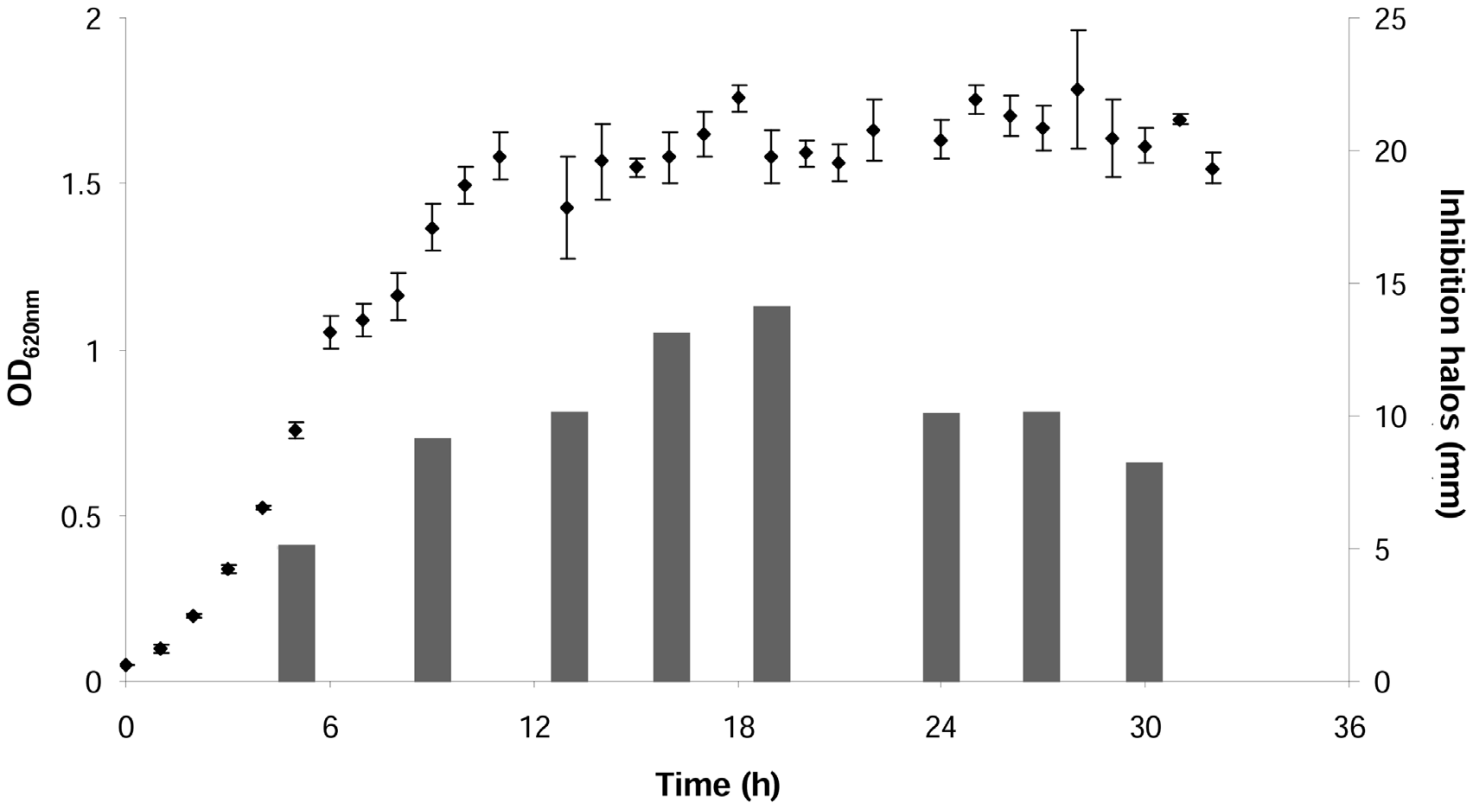
Growth of strain KM432Bz and bacteriocin production in MRS broth. Antimicrobial activity of cell-free supernatants is evaluated by radial diffusion assay against *Lact. sakei* subsp. *sakei* CIP 103139 and measure of the inhibition halos (▪). For the inhibition halos, standard deviation values are less than 2% and are not indicated. Values of optical density (⧫) are presented as the mean of three independent experiments with standard error of the mean.

Antibacterial activity of bacteriocin KM432Bz was detected in culture supernatants from 5 hours post-inoculation (exponential phase) until late stationary phase, using the highly sensitive strain *Lact. sakei* subsp *sakei* (Fig. 1). Maximum antibacterial activity (inhibition zones of 13–14 mm) was revealed in early stationary phase (16–19 hours after inoculation), followed by slow decrease along middle and late stationary phase.

### Purification of Bacteriocin KM432Bz

**Figure 2:**
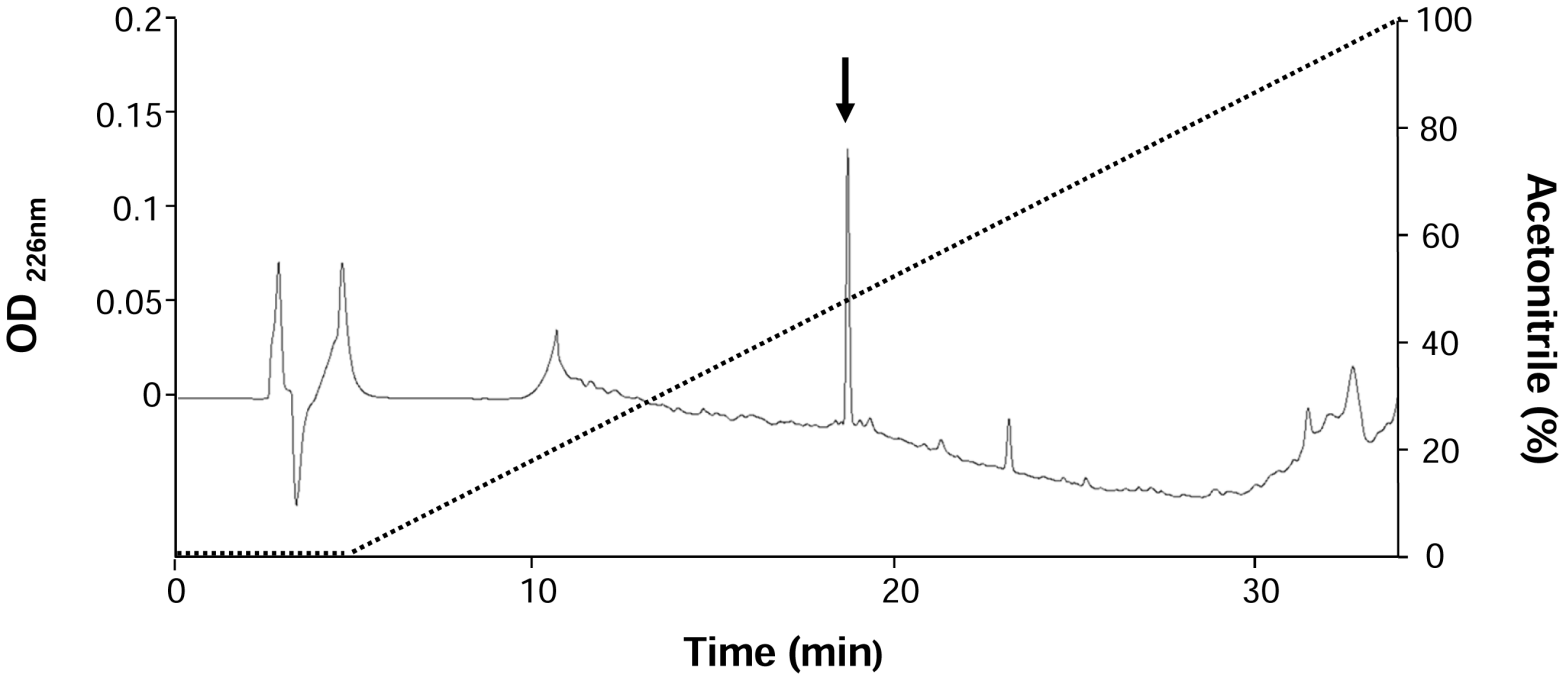
Reversed-phase HPLC elution profile of the 40% isopropanol Sep-Pak fraction. Separation was performed on an ODS2 Inertsil column under a gradient of 0 to 100% acetonitrile in 0.1% aqueous trifluoroacetic acid (dashed line). Arrow indicates the active fraction against *Lact. sakei* subsp. *sakei* CIP 103139 and *L. ivanovii* subsp. *ivanovii* CIP 78.42.

Purification of bacteriocin KM432Bz was guided by monitoring at each step the inhibitory activity against *Lact. sakei* subsp. *sakei* CIP 103139. The SepPak C18 fraction corresponding to 40% isopropanol, which was the most active against *Lact. sakei* subsp. *sakei*, was used for further purification by RP-HPLC (Fig. 2). Peak-associated fractions were hand-collected and tested for activity against *Lact. sakei* subsp. *sakei* and *Listeria ivanovii* subsp. *ivanovii* CIP 78.42. Only one peak retained antimicrobial activity on sensitive strains, with an inhibition zone of 14 mm against *L. ivanovii* subsp. *ivanovii*. Separation of bacteriocin KM432Bz by SDS-PAGE yielded a single peptide band of 3–4 kDa active against *Lact. sakei* subsp. *sakei* (data not shown).

### Determination of the Amino Acid Sequence of the Bacteriocin KM432Bz

**Figure 3:**
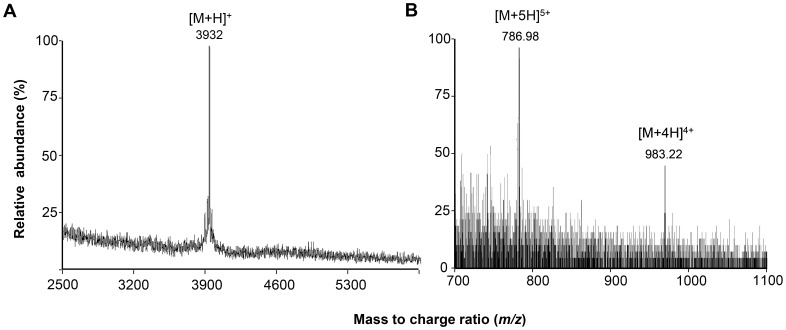
Mass spectrometry analysis of leucocin B-KM432Bz. (a) MALDI-TOF MS spectrum of the purified leucocin B-KM432Bz showing a single [M+H]^+^ ion at *m/z* 3932; (b) ESI-MS spectrum of the RP-HPLC fraction containing leucocin B-KM432Bz that exhibits [M+5H]^5+^ and [M+4H]^4+^ ions.

Purified bacteriocin KM432Bz was analysed by MALDI-TOF MS in positive linear mode and ESI-MS. MALDI-TOF MS revealed a single [M+H]^+^ ion at *m/z* 3932 (Fig. 3a), while ESI-MS showed two ions [M+5H]^5+^ and [M+4H]^4+^ at *m/z* 786.98 and 983.22 respectively (Fig. 3b), corresponding to a monoisotopic mass of 3928.78 Da. This is in agreement with the mass range of the active peptide detected in the SDS PAGE overlay.

**Figure 4:**
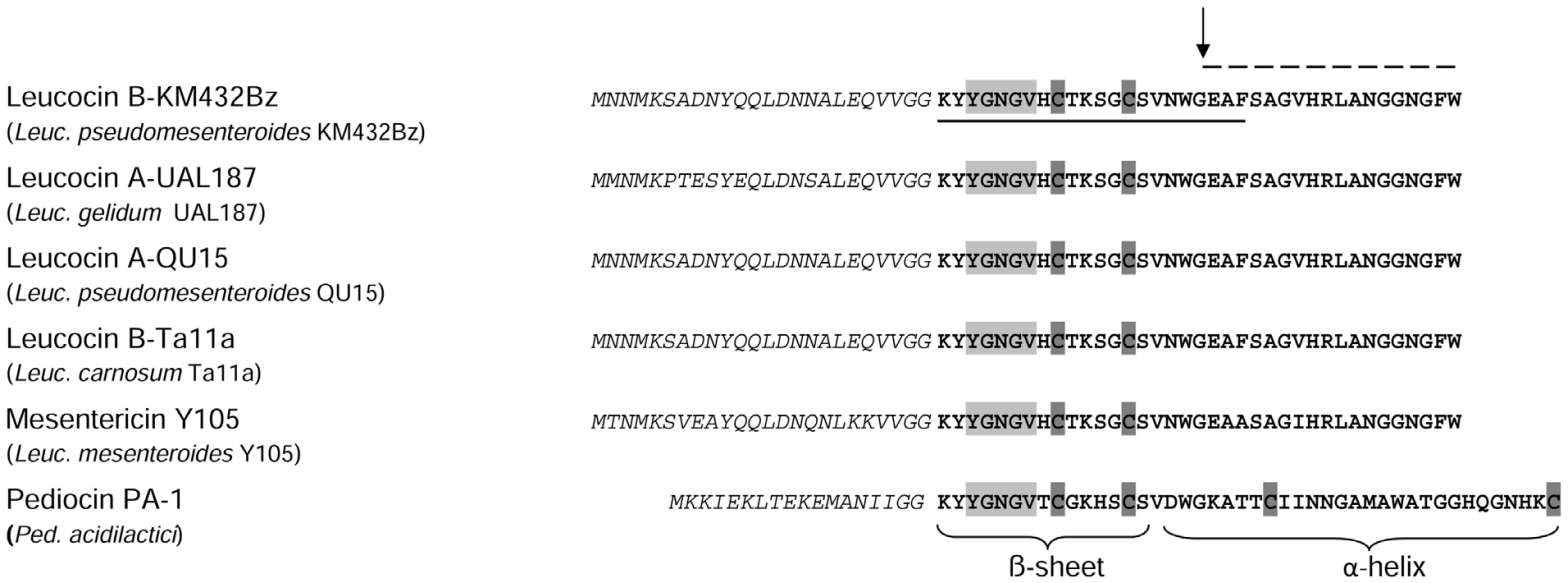
Alignment of amino acid sequences of class IIa bacteriocin precursors related to leucocin B-432Bz. Amino acid sequence of leucocin B-KM432Bz precursor (this study) was aligned class IIa bacteriocin precursors from *Leuconostoc*: leucocin A-UAL187, leucocin A-QU15, leucocin B-Ta11a, mesentericin Y105 and pediocin PA-1 from *Ped. acidilactici* (UniProtKB database accession numbers P34034, D7UPI7, Q53446, P38577, and P29430). Bold letters show the sequence of the mature bacteriocin and italics indicate the sequence of the leader peptide. The sequence of the leader peptide and the identification of leucine residue of leucocin B-KM432Bz were obtained by translation of the nucleotide sequence. Light grey background highlights the typical class IIa bacteriocin consensus sequence. Cysteines involved in the formation of the disulfide bridges are shown on dark grey background. The amino acid sequence of leucocin B-KM432Bz determined by Edman degradation is underlined. The amino acid sequence obtained by MS/MS fragmentation is indicated by a broken line. Arrow indicates the GluC endoproteinase cleavage site.

Edman degradation analysis revealed a 22-residue N-terminal sequence (Fig. 4), but no further sequence could be obtained. Subsequently, the sequence of the purified bacteriocin KM432Bz was further analysed by mass spectrometry, following reduction, alkylation, and digestion with GluC endoproteinase. Two peptide fragments corresponding to the Lys1-Glu20 N-terminal and the Ala21-Trp37 C-terminal regions were purified and analysed by micro-LC-MS/MS. A combined analysis of the results from Edman degradation and mass spectrometry revealed the full amino acid sequence of the bacteriocin (Fig. 4). However, an incertitude remained concerning the residue at position 29, as the isobaric residues leucine and isoleucine cannot be distinguished unambiguously by LC-MS/MS. Residue 29 was finally assigned to leucine, by translation of the gene sequence (see below). Therefore, taken together the data indicate that the active bacteriocin KM432Bz is a 37-residue peptide with a calculated average molecular mass of 3931.34 Da.

BLAST analysis of the amino acid sequence determined revealed 100% identity to the sequence of class IIa bacteriocins, leucocin A-UAL187 from *Leuconostoc gelidum* [28], leucocin A-QU15 from *Leuc. pseudomesenteroides* [29] and leucocin B-Ta11a from *Leuc. carnosum* [30], as shown in Fig. 4. The consensus sequence YGNGV, as well as the two cysteines involved in a disulfide bridge, which are typical of class IIa bacteriocins, are found along the sequence of bacteriocin KM432Bz (Fig. 4). This sequence differs only by two amino acid substitutions at positions 22 and 26 from that of mesentericin Y105, another class IIa bacteriocin isolated from *Leuc. mesenteroides* (Fig. 4).

### Analysis of the Gene Cluster Encoding Leucocin B-KM432Bz

**Figure 5:**
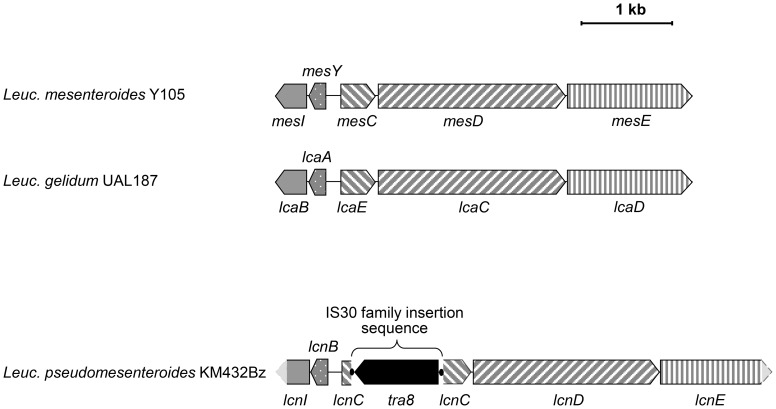
Gene clusters involved in the production of class IIa bacteriocins from *Leuconostoc*. The gene clusters are those of mesentericin Y105 produced by *Leuc. mesenteroides* Y105 [31] (Genbank accession number X81803), leucocin A-UAL187 produced by *Leuc. gelidum* UAL187 [35] (Genbank accession numbers M64371 and L40491) and leucocin B-KM432Bz produced by *Leuc. pseudomesenteroides* KM432Bz (this study, Genbank accession number JQ904469). Open reading frames belonging to the gene clusters are indicated by grey arrows: genes encoding the bacteriocin precursors (*mesY*, *lcaA*, *lcnB*, dotted arrows), genes encoding immunity proteins (*mesI*, *lcaB*, *lcnI*, plain grey arrows), genes encoding proteins of unknown function (*mesC*, *lcaE*, *lcnC,* arrows with right-inclined stripes), genes encoding ABC-transporters (*mesD*, *lcaC*, *lcnD,* arrows with left-inclined stripes), genes encoding secretion accessory proteins (*mesE*, *lcaD*, *lcnE,* vertically striped arrows). Broken lines in light grey at both ends of the *Leuc. pseudomesenteroides* KM432Bz gene cluster indicate that the 3′ end of *lcnI* and *lcnE* have not been sequenced. An IS30-family insertion sequence containing a *tra8* gene encoding a transposase (black arrow) interrupts the sequence of *lcnC* and is delimited by terminal inverted sequences (small black circles).

The nucleotide sequence of the bacteriocin KM432Bz gene cluster was aligned with the partial leucocin gene clusters in Genbank (data not shown), using Genbank accession number M64371 [1088 bp-leucocin A (*lcnA*) and immunity protein gene from *Leuc. gelidum* UAL187, the amino acid sequence of which will be called leucocin A-UAL187 below], L40491 [4300 bp-leucocin A ATP-dependent translocator (*lcaC*) and secretion protein (*lcaD*) genes from *Leuc. gelidum* UAL187], AB499610 [780 bp-leucocin A gene encoding LccA (*lccA*) from *Leuc. pseudomesenteroides* QU15, the amino acid sequence of which will be called leucocin A-QU15 below], and S72922 [804 bp-leucocin B and immunity protein genes from *Leuc. carnosum* Ta11a, the amino acid sequence of which will be called leucocin B-Ta11a below]. This alignment permitted to design primers specific to different regions of the gene cluster (Table 2). PCR amplification and sequencing allowed assembly of a 5.5 kb-sequence containing the gene cluster involved in the production of bacteriocin KM432Bz (Genbank accession number JQ904469, Fig. 5).

At first the leader peptide of bacteriocin KM432Bz was identified from translation analysis of the gene cluster sequence and showed to have an identical amino acid sequence to that shared by leucocin B-Ta11a and leucocin A-QU15 (Fig. 4), the latter having also been isolated from a *Leuc. pseudomesenteroides* strain. Because leucocin A from *Leuc. gelidum* UAL-187 and leucocin B from *Leuc. carnosum* Ta11a have been described long before the peptide from strain QU15, we assume that the bacteriocin from *Leuc. pseudomesenteroides* QU15 has been wrongly assigned to leucocin A and should have been called leucocin B. We therefore named the bacteriocin leucocin B-KM432Bz. Since the gene nomenclature for bacteriocins is heterogeneous, the genes were named here following the nomenclature used for the mesentericin Y105 gene cluster [31], [32], which is the most complete gene cluster available in databases (GenBank accession number X81803) for such class IIa bacteriocins, using *lcn* as an abbreviation for leucocin. The general organization of the gene cluster encoding leucocin B-KM432Bz is shown in Fig.5 compared to those of mesentericin Y105 and leucocin A-UAL187. The gene clusters follow the same pattern of organization. Two genes encode the precursors (*mesY*/*lcaA*/*lcnB*) and the putative immunity proteins (*mesI*/*lcaB*/*lcnI*) of mesentericin Y105, leucocin A-UAL187 and leucocin B-KM432Bz. The sequence of the immunity protein LcnI is identical to those of leucocins A-UAL187 and B-Ta11a, and shares 71% identity with MesI, the immunity protein for mesentericin Y105. Heading to the opposite direction, two genes encoding the ATP-binding cassette (ABC) transporters (*mesD*/*lcaC*,/*lcnD*) and secretion accessory proteins (*mesE*/*lcaD*/*lcnE*), have been shown to encode the dedicated export system in the case of mesentericin Y105 [32]. LcnD shares 98.5% and 96.5% identities with LcaC and MesD from leucocin A-UAL187 and mesentericin Y105, respectively, while LcnE shares 96% and 91% identities with LcaD and MesE from leucocin A-UAL187 and mesentericin Y105, respectively.

An additional gene of unknown function (*mesC*/*lcaC*/*lcnC*), for which the deduced amino acid sequences share 71% identity, is present between the genes encoding the bacteriocin precursor and the ABC transporter. In *Leuc. pseudomesenteroides* KM432Bz the sequence of *lcnC* is interrupted by a 1027-bp insertion sequence (Fig. 5), which presents typical features of a member of the IS30 family: direct dinucleotide repeats (TT, repeated twice), a 27-bp inverted repeat at each end of the IS and an ORF encoding a 305-residue Tra8 transposase.

### Antimicrobial Activity of Leucocin B-KM432Bz

The inhibitory activity of leucocin B-KM432Bz was examined against a range of Gram-positive bacteria and *Klebsiella pneumoniae,* using the radial diffusion assay (Table 1). Leucocin B-KM432Bz did not impair the growth of *Kl. pneumoniae*, but inhibited the growth of phylogenetically related strains of lactic acid bacteria belonging to the genera *Lactobacillus* or *Weissella,* but also of several strains of *Leuconostoc*. Leucocin B-KM432Bz was also active against a few enteric pathogens, such as *Ent. faecium* HKLHS and *Enterococcus faecalis* CIP 103015, and one respiratory tract pathogen, *Streptococcus pneumoniae* CIP 102911 (Table 1).

Leucocin B-KM432Bz inhibited the growth of five strains of *Listeria* (*L. innocua* CIP 80.11, *L. ivanovii* subsp. *ivanovii* CIP 78.42, *L. monocytogenes* CIP 82.110, CIP 107776, and AML73, Table 1). We determined the MICs of leucocin B-KM432Bz over a range of sensitive strains, including three *Listeria,* among which the human pathogen *L. monocytogenes* CIP 82.110 (Table 3), and compared the values to those we obtained for pediocin PA-1 (Table 3). The MICs were in comparable ranges for both bacteriocins as concerns strains closely related to the producer, such as *Leuc. lactis* CIP 102422, *Lact. sakei* subsp. *sakei* CIP 103139, and the pathogen *Ent. faecium* HKLHS. MIC values confirmed the particular sensitivity of *Lact. sakei* subsp. *sakei* CIP 103139 to leucocin B-KM432Bz, but also to pediocin PA-1, as the MIC was the lowest obtained along this study (8 nM). For the three tested *Listeria* strains, the MIC values were much lower for leucocin B-KM432Bz (16–64nM) than for pediocin PA-1 (256–1024 nM). In the case of *Strep. pneumoniae* CIP102911, the MIC for leucocin B-KM432Bz was 256 nM, while pediocin PA-1 hardly inhibited its growth.

**Table 3:** Minimal Inhibitory Concentrations^a^ (nM) of purified leucocin B-KM432Bz and pediocin PA-1 on sensitive strains.

Target strain	Leucocin B-KM432Bz	Pediocin PA-1^b^
*Enterococcus faecium* HKLHS	256	256
*Lactobacillus sakei* subsp. *sakei* CIP 103139	8	8
*Leuconostoc lactis* CIP 102422	1024	256
*Leuconostoc mesenteroides* subsp. *mesenteroides* CIP 102305	128	16
*Listeria innocua* CIP 80.11	64	256
*Listeria ivanovii* subsp. *ivanovii* CIP 78.42	32	>1024
*Listeria monocytogenes* CIP 82.110	16	512
*Streptococcus pneumoniae* CIP 102911	256	>1024

a The MIC is defined as the lowest bacteriocin concentration for which no growth could be observed,

b Pediocin used in this study was purchased from Sigma (P0098).

### Insights into the Mode of Action of Leucocin B-KM432Bz

Mesentericin Y105, for which the amino acid sequence differs from that of leucocin B-KM432Bz only by two residues, depends on σ^54^ regulated-transcription in the target cell to exert its antimicrobial effect [33]. Moreover, it has been shown the antimicrobial activity of classIIa bacteriocin over sensitive strains required the presence of the EII^t^_Man_ mannose permease whose expresssion is regulated by σ^54^ [34]. We performed antimicrobial assays using leucocin B-KM432Bz over a strain of *L. monocytogenes* for which the *rpoN* gene encoding the σ^54^ transcription factor had been inactivated. This strain (EGDe *rpoN* ^-^) showed to be resistant to leucocin B-KM432Bz, while the wild-type isogenic EGDe strain (CIP 107776) retained sensitivity to leucocin B-KM432Bz (Table 1). We also performed antimicrobial assays using leucocin B-KM432Bz over *L. monocytogenes* AML73 and the corresponding mutants for subunits AB (Δ*manL*) and C (Δ*manM*) of the EII^t^_Man_ mannose permease. While the wild-type AML73 strain and the Δ*manL* mutant were sensitive to leucocin B-KM432Bz, the Δ*manM* mutant was resistant to the bacteriocin (Table 1). Therefore, the antimicrobial effect of leucocin B-KM432Bz over *Listeria* requires expression of compound(s) regulated by the σ^54^ transcription factor, and it also requires expression of subunit C, but not subunits AB, of the EII^t^_Man_ mannose permease.

## Discussion

While boza has been reported to be a prolific source of bacteriocin-producing bacteria [4]–[6], [19], [20], this work presents the first thorough characterization of a class IIa bacteriocin isolated from this fermented beverage. Class IIa bacteriocins also called “pediocin-like” bacteriocins are cationic antimicrobial peptides with a molecular mass below 5.5 kDa, which share the ability to inhibit the growth of *Listeria* strains [12]. They are generally produced as an inactive precursor carrying an N-terminal leader peptide and are exported by an ABC transporter. The proteolytic domain of the ABC transporter ensures cleavage of the N-terminal leader peptide to yield the active bacteriocin [34]. The nomenclature of class IIa bacteriocins is somewhat confusing. All being produced by *Leuconostoc* strains, the amino acid sequence of mature forms of leucocin A-UAL187, leucocin A-QU15, leucocin B-Ta11a, and leucocin B-KM432Bz are identical, but the N-terminal leader peptide diverges for leucocin A-UAL187. Therefore, leucocin B-KM432Bz represents the third occurrence of an identical active peptide found in *Leuconostoc* strains.

The gene clusters encoding leucocins have not been studied thoroughly, excepting those encoding leucocin A-UAL187 [35] and mesentericin Y105, which is another class IIa bacteriocin produced by a *Leuc. mesenteroides* strain for which the amino acid sequence differs from that of leucocins A-UAL187, A-QU15, B-Ta11a and B-KM432Bz only by two residues [31], [32]. We describe here the first complete gene cluster for a type B leucocin. In *Leuc. pseudomesenteroides* KM432Bz, the gene cluster encoding leucocin B-KM432Bz was found to show a similar organization to those found in the strains producing mesentericin Y105 and leucocin A-UAL187 (Fig. 5). Two groups of genes encode the leucocin precursor (*lcnB*) and an immunity protein (*lcnI*) on the one hand and the dedicated export machinery on the other hand, which consists of an ABC-transporter encoded by *lcnD* and a secretion accessory protein encoded by *lcnE*. The export system would be conserved in this bacteriocin family, as the sequences of both the ABC transporter and accessory protein from leucocin B-KM432Bz are highly similar to those reported for leucocin A-UAL187 and mesentericin Y105 with amino acid sequence identities higher than 91%.

An additional gene (*mesC/lcaE*/*lcnC*) is found between the genes encoding the bacteriocin precursor and the ABC transporter. The sequence of *lcnC* in *Leuc. pseudomesenteroides* KM432Bz is interrupted by an insertion sequence of the IS30 family [36]. The IS30 family is widespread in lactic acid bacteria, and has already been reported in several species of *Leuconostoc*. The Tra8 transposase sequence from the leucocin B-KM432Bz gene cluster shows only one amino acid substitution compared to that of the transposase encoded in the genome of *Leuconostoc citreum* KM20. Despite inactivation of *lcnC* due to an interruption of the coding sequence by an IS, leucocin B-KM432Bz was isolated from strain KM432Bz. Therefore, *lcnC* does not play an essential role in the production of leucocin B-KM432Bz. A similar result has been reported in the gene cluster encoding production of leucocin A-UAL187. Indeed, a frameshift mutation in *lcaE* did not affect leucocin A-UAL187 production, while deletions of *lcaC* and *lcaD* resulted in loss of bacteriocin production [35]. While no putative function has been proposed for *mesC/lcaE*, it is nevertheless present in at least three gene clusters involved in class IIa bacteriocin production (mesentericin Y105, leucocin A-UAL187, leucocin B-KM432Bz).

One characteristic of class IIa bacteriocins is their potent inhibition of *Listeria*. This was confirmed for leucocin B-KM432Bz, which showed potent inhibitory activity against the three strains of *Listeria* tested (*L. innocua* CIP 80.11, *L. ivanovii* subsp. *ivanovii* CIP 78.42, and *L. monocytogenes* CIP 82.110). We determined the MIC values, defined as the lowest concentration of bacteriocin inhibiting growth, of leucocin B-KM432Bz over sensitive strains, in comparison with those of pediocin PA-1. Pediocin PA-1, produced by *Ped. acidilactici* is the most extensively studied bacteriocin of class IIa [37], which are also called “pediocin-like” bacteriocins. This bacteriocin has shown inhibition of *L. monocytogenes* in several foods, such as cheese, frankfurters or sausages [34]. MIC values confirmed the particular sensitivity of *Lact. sakei* subsp. *sakei* CIP 103139 to leucocin B-KM432Bz, but also to pediocin PA-1, as the MIC was the lowest obtained in this study (8 nM). Against *Listeria* strains, leucocin B-KM432Bz was 4- to 32-fold more active than pediocin PA-1. Indeed, the MICs were in the range 16–64 nM for leucocin B-KM432Bz, while pediocin PA-1 was active only at 256–1024 nM over the same *Listeria* strains. Although at higher concentrations, a similar observation can be made for inhibition of another pathogen, *Strep. pneumoniae* CIP 102911.

Class IIa bacteriocins exert their antimicrobial effect by permeabilizing the cytoplasmic membrane of the target cell [12], [34]. They are unstructured in water but become organized in two structural regions in membrane-mimicking environments. The conserved, ß-sheet, positively charged N-terminal region would allow initial binding of the peptide at the target cell surface, presumably through electrostatic interactions, and would be responsible for the specific inhibition of *Listeria* strains. The more variable in terms of sequence, yet structurally conserved α-helical C-terminal region would interact specifically with their receptor, which has been proposed to be subunit C of the EII^t^_Man_ mannose permease [34], [38]. Indeed, inactivation of the *rpoN* gene encoding the σ^54^ transcription factor induces resistance of *L. monocytogenes* to mesentericin Y105 [33]. The σ^54^ transcription factor triggers transcription of the gene encoding the EII^t^_Man_ mannose permease [39]. Disruption of the genes encoding the EII^t^_Man_ mannose permease in *L. monocytogenes* and *Ent. faecalis* confered resistance to class IIa bacteriocins, and heterologous expression of subunit C of the permease rendered *Lactococcus lactis* sensitive to class IIa bacteriocins [34]. The *Listeria* strain, for which the *rpoN* gene encoding the σ^54^ transcription factor is inactivated, as well as the *Listeria* mutant for the gene encoding subunit C of the EII^t^_Man_ mannose permease (Δ*manM*), proved to be resistant to leucocin B-KM432Bz, while a mutant for subunits AB retained sensitivity (Table 1). This is consistent with previously reported results for other class IIa bacteriocins [38]. Therefore, like other class IIa bacteriocins, leucocin B-KM432Bz uses subunit C of the EII^t^_Man_ mannose permease as a receptor to exhert its inhibitory effect on target strains, and presumably this is the case also for leucocins A-UAL187 and B-Ta11a, which share the same amino acid sequence.

Leucocin B-KM432Bz and pediocin PA-1 differ mainly in the C-terminal region, which is longer and contains an additional disulfide bond in the case of pediocin PA-1 (Fig. 4). A few studies compared efficiency of class IIa bacteriocins displaying or not this second disulfide bridge [40]–[42]. They conclude that class IIa bacteriocins with a second disulfide bond are more stable at 37°C than at 25°C, while this correlates with better efficiency against some target strains including *Listeria* strains. This result is in apparent contradiction with those of this study, for which the MIC of leucocin B-KM432Bz over three *Listeria* strains is lower than that of pediocin PA-1 at 37°C. However, the definition of MIC used in our work is the lowest bacteriocin concentration for which no growth of the target strain could be observed, while the above-mentioned studies define MIC as the concentration of bacteriocin that inhibits growth of the indicator strain by 50% [40]–[42], which in most cases corresponds rather to the definition of IC_50_. Therefore, the divergence in estimating the efficiency of both bacteriocins in previous studies and our work explains that we do not draw the same conclusions from similar experiments. We infer from our results that leucocin B-KM432Bz is more potent than pediocin PA-1, and our results are strengthened by the fact that they were confirmed over three target *Listeria* strains, including the pathogen *L. monocytogenes*.

Several studies have demonstrated the potential of class IIa bacteriocins to be used as a food preservative, because they can inhibit the growth of both spoilage and pathogen bacteria. Pediocin PA-1 has been used successfully to reduce the growth of *Listeria* in several foods, sometimes combined with other methods such as post-packaging pasteurization or vacuum packaging. Pediocin is commercially available from Quest International as ALTA2341 [16], [34], [43]. On the other hand, inoculation of the leucocin A-UAL187 producer *Leuc. gelidum* to meat has been shown to delay the spoilage of meat, and reducing putrid odors normally associated with the development of *Lact. sakei* [34]. Therefore, leucocin B-KM432Bz harbours a good potential to be used as food preservative or agent lengthening shelf storage, since, at lower concentrations than pediocin PA-1, it clearly inhibits the pathogen *L. monocytogenes* and food spoilage bacteria.
